# Development and validation of a potential assessment inventory for assessing EFL teachers’ ecological agency

**DOI:** 10.1186/s40468-022-00190-5

**Published:** 2022-09-08

**Authors:** Masoumeh Ghamoushi, Zohre Mohammadi Zenouzagh, Mohammad Hashamdar

**Affiliations:** Department of English Translation and Teaching, Karaj Branch, Islamic Azad University, Karaj, Iran

**Keywords:** Ecological agency, Iterational, Practical-evaluative, Projective, Teacher agency

## Abstract

Teacher agency as an influential factor in teacher professionalism has recently gained global inquiry in the EFL context. However, no valid instrument has ever been designed to evaluate EFL teachers’ ecological agency. This gap prompted the researchers of the current study to develop and validate a questionnaire to assess EFL teachers’ ecological agency. In the first phase, a comprehensive review of the literature and semi-structured interviews were conducted to determine the underlying components of the teacher ecological (TEA) questionnaire based on Priestley et al.’s (*Flip the System* 134–148, 2015) ecological agency model. In the second phase, the newly developed TEA questionnaire including 40 items was subjected to reliability and validity issues. Therefore, it was piloted with 222 Iranian EFL teachers selected through non-probability convenience sampling. The Cronbach alpha results confirmed an acceptable reliability index (.858). The results of factor analysis revealed that the number of items was reduced to 37 and indicated that the data on teacher ecological agency loaded on 3 components: iterational (9 items), practical-evaluative (14 items), and projective (10 items). In addition, the structural equation modeling (SEM) results confirmed that the model enjoyed sound psychometric properties. The upshots of the current study have undoubtedly significant implications for teacher educators and teaching practitioners.

## Introduction

Human agency is recently explicated in different professional settings. Some researchers have speculated how it develops and is moderated by contextual elements. Agency is broadly defined as a human’s active participation in forming realities which is a critical condition for effective functioning in every aspect of life, especially in the workplace (Leijen et al., 2020). To be agentic, not only individuals are required to be capable of making an accountable sound judgment about the significance of their motives when they act out, but also they should be able to evaluate whether they have met the objectives they have set. In addition, the professional agency encourages individuals to make principled decisions and affect their profession and professional identity, and it is regarded as a phenomenon that can be trained (Edwards, 2015).

Recent years have witnessed a growing interest in the concept of agency in various educational settings in general and in teacher education in particular as a crucial factor that affects teachers’ professional development. Teachers are expected to take intentional and agentic actions and make decisions in their professional contexts which leads to significant changes. That is, teachers are required to act by employing their contexts instead of being in their contexts via making efforts and implementing the available resources (Teng, 2019). In other words, agentic teachers have a voice and are empowered to negotiate and overcome the constraints imposed by the policy in their everyday teaching practice. Empirical research indicates that English teachers apply agency in several ways such as resistance, modification, and compliance in their own contexts. Therefore, it is essential to examine teachers’ agency in different contexts (Le et al., 2021).

Agency has been examined from different points of view in education, such as social cognitive theory (Bandura, 1989), positioning theory (Davies, 2000), and the ecological approach (Priestley et al., 2015). The ecological approach to agency which is the focal point of this study, relies on Emirbayer and Mische’s (1998) chordal tried agency that defines agency as a “temporally embedded process of social engagement, informed by the past, oriented toward the future and acted out in the present” (p. 963). Later on, Priestley et al. defined teacher agency as “a progressive and situated achievement, that is the result of the interaction between iterational, practical-evaluative, and projective dimensions” (Priestley et al., 2015, p. 29). These three dimensions are vital in defining teacher agency. The iterational dimension comprises a teacher’s life history accompanied by professional biographies. The practical-evaluative dimension includes cultural (ideas, values, and beliefs), structural (social structures, roles, and relationships), and material (resources and physical environment) aspects. The projective dimension consists of long-term and short-term activities and goals (Kayi-Aydar et al., 2019).

In the present study, Priestley et al.’s ecological agency framework was applied because it is a comprehensive framework for some reasons. First of all, it seems to be the first framework that tackles the concept of teacher agency from the ecological point of view. Notably, it considers the construct of teacher agency as an emergent construct. Moreover, this is not only a theoretical framework but also an analytic instrument that can be used for empirical studies, and their results revealed the practicality of this framework (Zhang & Wright, 2017).

A body of burgeoning studies investigated teacher agency from various standpoints such as the impact of teacher agency on teachers’ professional development and the general quality of education (Lai et al., 2016; Ruan, 2018), the effect of teacher reflection on teacher agency (Jones & Charteris, 2017; Reichenberg, 2022), the interplay between teacher agency and teacher identity (Buchanan, 2015; Connolly et al., 2018), teacher agency and identity commitment (Tao & Gao, 2017), the effect of curriculum reform on teacher agency (Poulton, 2020; Ruan et al., 2020), and the role of teacher agency in inclusive education (Lyons et al., 2016; Naraian, 2014; Naraian & Schlessinger, 2018; Themane & Thobejane, 2019). The data collection methodologies utilized in these studies for the teacher agency construct are mainly based on interviews, reflective essays or diaries, classroom observation, portfolios, focused group discussions, document analysis, and teachers’ narratives. Therefore, based on the comprehensive literature review and despite existing appeal in this construct, the researchers of the present study did not find any valid and reliable questionnaires for assessing teachers’ agency.

Undoubtedly, cultivating teacher agency results in empowered individuals who have a voice and act agently in their everyday professional practice and understanding teacher agency is regarded as a critical aspect of educational research which is an under-research topic. However, researchers and practitioners may be confronted with the lack of an instrument with sound psychometric properties to measure teachers’ ecological agency. Thus, designing and validating an instrument that can assess teachers’ level of agency is essential. To bridge this research gap, the current study aimed at developing and validating a potential questionnaire for assessing EFL teachers’ agency based on Priestley et al.’s (2015) ecological model.

## Literature review

### Teacher agency

Scholars have used the concept of teacher agency to explain teachers’ agentic potentiality to make effective decisions in pedagogical practices. Teacher agency deals with teachers’ capacity to create and conduct pedagogical changes, and apply and adjust their activities in educational settings (Sang, 2020). Teachers are regarded as critical agents influencing the enhancement of educational policy, applying policy in their teaching practice, and providing learners with learning opportunities. Hence, educational researchers have focused on teacher agency as a construct playing a crucial role in educational development (Li & Ruppar, 2021).

Generally, agency is defined as a “socioculturally mediated capacity to act” (Ahearn, 2001, p. 130), which is regarded as an integral element of teachers’ professional development, wherein they are supposed to apply power, take action, and make a change. The process of achieving agency is not linear, it is a constant broad process that is dynamic, multifaceted, and conditional (Ruan, 2018). Teachers have complex duties, handling more than one thing simultaneously. They are demanded to adjust to their teaching context, interact with both their colleagues and parents, decide about their teaching practices, and have reciprocal understandings. By making these efforts, teachers can provide a positive and encouraging environment for their students, their colleagues, and themselves in their professional setting. This complex relationship between the aforementioned issues portrays the multifaceted nature of teacher learning.

### Experimental background

Several empirical studies support the effect of teachers’ agency on their professional learning and development. In a study, Lai et al. (2016) explored how teacher agency affects their professional learning in cross-cultural teaching settings. The results of interviews with Chinese language teachers revealed that teacher agency fluctuated in various dimensions of professional learning. Different factors such as power relations, social suggestions, imposed identity, teachers’ professional status, and their social roles and positioning have a part in shaping teacher agency. The finding also indicated that school cultures and structures that appreciate and share various educational practices and regulate teachers’ professional identity can promote teachers’ agency. In another study, by applying a qualitative approach and implementing qualitative data collection instruments such as interviews, classroom observation, artifacts, and living graphs, Ruan (2018) found that (1) EFL teachers create and recreate their agency through the influences of the past, involvement with the present, and orientation toward the future. (2) Achievement of agency can be facilitated through self-regulation and reflection. (3) Teachers’ agency can be supported or confined by their context.

In addition, scholars investigated the development of teachers’ agency through reflective practice. For instance, Jones and Charteris (2017) addressed the effect of critical reflection on teachers’ professional learning. They believed that novice teachers’ agency is enhanced in transformative professional learning depicted through partnerships, which offer decision-making and critical reflection capacities. Similarly, in a recent study, Reichenberg (2022) investigated the effect of the reflective process and lesson video on teachers’ agency in the literacy coaching context. Results indicated that teachers’ actions during reflection resulted in teachers’ agency practice which was noticing, regarding learners’ viewpoints and inferring. These teachers’ acts support educational changes to promote instructional scaffolding and boost learner engagement and learning in complicated settings.

Regarding the relationship between teacher agency and identity, several researchers claimed that teachers’ identity can affect their agency. Buchanan (2015) tried to understand how teachers make sense of their professional selves throughout the educational reform in California via conducting semi-structured interviews. In her study, she examined how teachers’ professional identity and agency were formed by reform settings and discourses. Moreover, she explored how teachers overcome and negotiate aiming to create a status for themselves not only in their institutes but also in the present national policy and reform atmosphere. By the same token, Tao and Gao (2017) explored the interplay of teacher agency and identity commitment to their professional growth during curriculum reform. The results of historical interviews and field notes indicated various actions and choices in professional courses. Teachers’ identity commitment affected their agentic choices and their particular way of enacting agency was mediated by their past experiences. Moreover, the incorporation of competing for professional identities can boost teachers’ sense of professional agency (Connolly et al., 2018).

Various studies have attempted to explore teachers’ agency in curricular reform. Ruan et al. (2020) for example, examined English department teachers’ agency in the context of the classroom by adopting journal entries, semi-structured interviews, and classroom observations. The thematic analysis of the data showed that teachers desired to enact their agency in different meaning-making attempts, in order to promote their teaching efficiency in the classroom. Moreover, this study speculated on the interplay between teachers’ recognized self-discrepancy and agency in their teaching practice in a curriculum reform atmosphere. In the same vein, Poulton (2020) conducted a case study to investigate Australian primary school teachers’ experiences in top-down and bottom-up curriculum reforms. The content analysis of the semi-structured interviews revealed some elements as promoters and demoters to teachers’ agency in planning and teaching the curriculum.

In the current climate of social justice and equity, a growing body of research (Lyons et al., 2016; Naraian, 2014; Naraian & Schlessinger, 2018; Themane & Thobejane, 2019) emphasizes the agentic role of teachers in providing inclusive education and equitable atmospheres for marginalized students. Naraian (2014) for instance utilized a situated concept of agency to discover teachers’ activities in several schooling discourses via observation, interview, and document analysis. The results indicated that teacher education discourses that benefit the policy of inclusion vs. exclusion are not sufficient to fulfill the complex requirements of permitting the practice of inclusivity. Likewise, analyzing the data collected through interviews, observations, and document analysis from eight teachers in South Africa, Themane and Thobejane (Themane & Thobejane, 2019) reported that teachers did their best and were resilient despite the lack of resources to perform inclusive education and showed eagerness to enact change. Moreover, in the case of collaboration with others, they achieved the desired outcomes.

## Theoretical framework

### Ecological approach to teacher agency

**Fig. 1:**
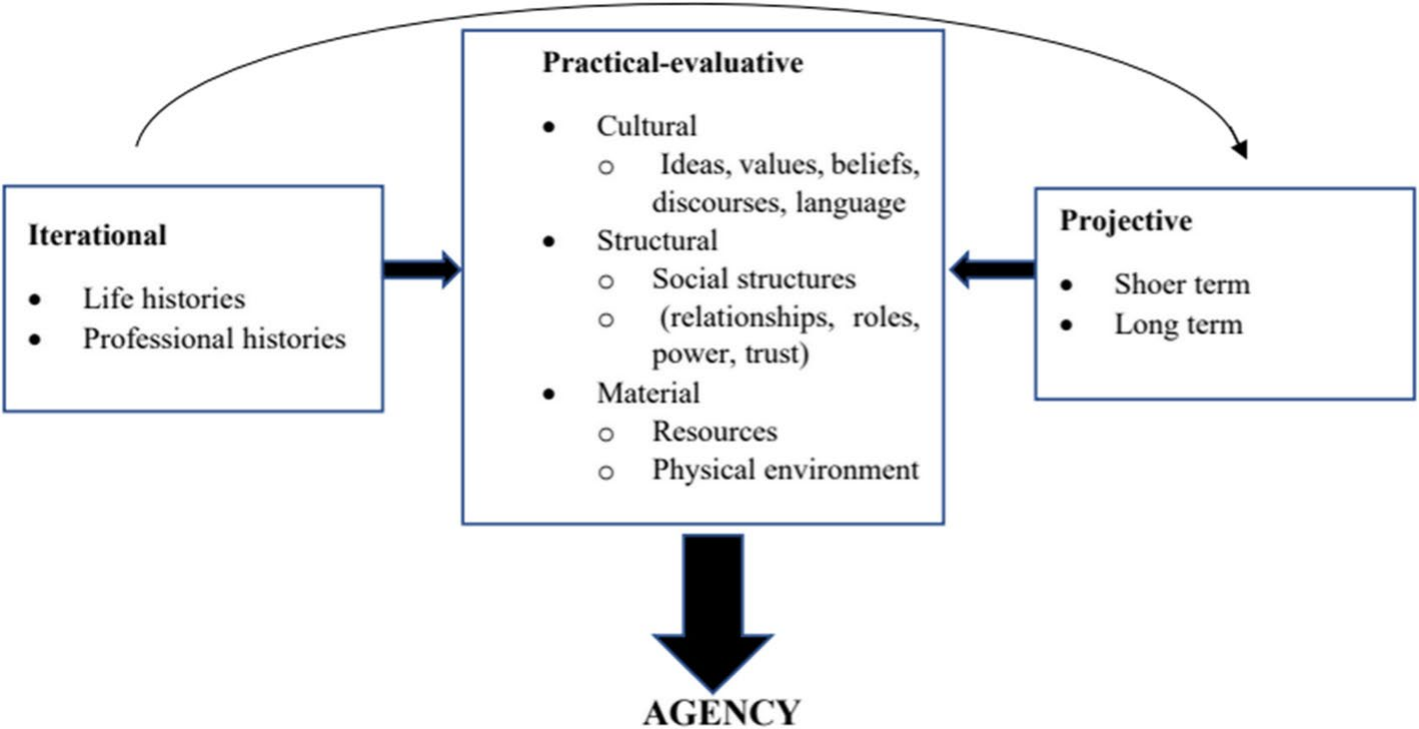
Ecological model of teacher agency (Priestley et al., 2015, p.30)

The researchers of the present study adopted the ecological approach to teacher agency as their main conceptual framework. Drawing on Emirbayer and Mische’s (1998) ecological perspective of teacher agency, Priestley et al. (2015) defined teacher agency as a time-based and situated achievement that was the result of the interaction among iterational, practical-evaluative, and projective dimensions as the three crucial dimensions of ecological teacher agency (Fig. 1).

As illustrated above, the three dimensions of ecological teacher agency are included in this model. The iterational dimension consists of teachers’ personal capacity, beliefs, and values that reflect teachers’ past experiences. This dimension can be developed by promoting teachers’ knowledge and skills and encouraging an innovative and questioning mindset. Everyday teaching practice, dialogic interactions with co-workers, and other professional engagements are influential as well in shaping teacher agency (Priestley et al., 2015).

The projective dimension of teacher agency deals with teachers’ work-related ambitions and their short- and long-term goals. These ambitions may be completely positive dealing with students’ development and resulting in protecting students’ interests or they may counteract. In both cases, teachers’ ambitions and goals have their roots in their beliefs, values, and experiences that permit creating actions consistent with the actor’s probable future trajectories (Priestley et al., 2015).

The practical-evaluative dimension makes a distinction between different contextual elements that affect teacher agency. That is to say, various structural contexts provide teachers not only with required conditions but also resources to achieve agency. The practical aspect reflects what is practically possible in the particular context and the evaluative aspects deal with how teachers evaluate the current issues and feasibilities for action in that situation. The achievement of agency relies on the availability of required resources that can be employed in the situation. This dimension includes cultural, material, and structural resources. Cultural resources are concerned with (1) teachers’ way of thinking, and understanding of the issue and the situation. It also deals with teachers’ inner dialogue (i.e., their own thinking) and outer dialogue (i.e., their conversations and interactions with others in a particular situation); (2) the available material and physical resources; and (3) teachers’ social interaction and relationships with others that support or hamper the achievement of agency (Priestley et al., 2015). Therefore, based on Priestley et al.’s ecological view, agency is something that can be achieved as the consequence of the everyday actions that teachers take and decisions that they make influenced by the various aspects of the present context and their past experiences and future objectives.

It is worth mentioning that the researchers of the current study have scrutinized and used all the abovementioned studies and frameworks to extract the components and items of the newly developed TEA questionnaire. As this literature review illustrates, although the aforementioned studies have been conspicuous endeavors to offer a thorough definition of the concept of teacher agency and explore this construct by implementing qualitative data collection instruments, none of them have developed and validated a questionnaire to assess EFL teachers’ ecological agency.

Because of this, the current study is the first attempt to design and validate a questionnaire with sound psychometric properties to evaluate teachers’ ecological agency in the context of English as a foreign language. This study sought to address the following questions:

- Q1. What are the underlying components of the Teacher Ecological Agency (TEA) questionnaire?
- Q2. What are the psychometric properties of the Teacher Ecological Agency (TEA) questionnaire?
- Q3. To what extent does the structural model of the Teacher Ecological Agency (TEA) questionnaire fit the hypothetical model generated by the relevant literature review?

## Method

### Participants

To collect the required data, two independent groups of participants took part in this study. In the initial phase, 20 male (15%) and female (85%) EFL teachers between the ages of 28 and 42 participated in semi-structured interviews through convenience sampling. The participants had a minimum of 6 years of teaching experience. As for their educational status, 5% of the participants were Ph.D. holders, 55% were Ph.D. candidates, 30% had MA and 10% had BA in English majors (TEFL, Translation, and English Literature). In the next phase, a total number of 222 male and female EFL teachers took part in this study. They were both male and female EFL teachers with various age ranges and teaching experience. The sample was selected based on convenience sampling. In other words, participants were invited to take part based on accessibility or opportunity (Best & Kahn, 2006). Table 1 below is a representation of these participants’ demographic features.

**Table 1:** Characteristics of participants in the second phase

Participants’ characteristics		Frequency
Age range	20–30	87
	31–40	105
	>40	30
Degree	B.A.	85
	M.A.	95
	Ph.D.	42
Major of study	TEFL	101
	Translation	68
	Literature	53
Teaching experience	1–5	32
	6–10	72
	11–15	89
	>15	29
Gender	Male	52
	Female	170
Total		222

For determining the sample size of the current study, Pallant’s (2016) approach to sample size estimation was followed. According to Pallant (2016), for determining the ideal number of participants to answer a questionnaire in the piloting phase, five cases per variable would be a good sample size. Since the Teacher Ecological Agency (TEA) questionnaire includes 40 items loaded on the three components of (1) iterational, (2) practical-evaluative, and (3) projective in Priestley et al.’s (2015) ecological agency model, a minimum sample size of 200 participants was required in this study.

### Instruments

The researchers of the current study carried out a comprehensive review of literature on teacher agency to determine the underpinning theoretical framework, conduct the semi-structured interview questions, identify the themes, and generate the items (e.g., Biesta et al., 2015; Emirbayer & Mische, 1998; Eteläpelto et al., 2015; Kayi-Aydar et al., 2019; Leijen et al., 2021;; Peña-Pincheira & De Costa, 2021; Priestley et al., 2015; Wallen & Tormey, 2019; Wang et al., 2021).

To achieve the purpose of this study, two instruments were utilized. The first one was a semi-structured interview including 7 questions (which are mentioned in the following part) and a 5-point Likert scale questionnaire including 40 items that address the characteristics of a teacher’s ecological agency. This questionnaire was developed by the researcher, getting help from different sources including a set of interview questions, and doing a comprehensive review of the related literature.

### Procedure

Having conducted a comprehensive review of the literature on teacher ecological agency, the researchers of the current attempt determined the theoretical framework which shaped the initial steps demanded in designing the questionnaire based on which the semi-structured interview questions were proposed. The following table represents the semi-structured interview questions, their functions, and sources.

As illustrated in Table 2, all of these questions take the three components of teachers’ ecological agency into account. Utilizing these questions, semi-structured interviews were conducted with 20 EFL teachers. Each interview lasted about 20 min, audio-recorded, transcribed, and coded applying NVivo software 10. These codes and themes were further used to generate the items of the questionnaire. This was done collaboratively by the two researchers and cross-checked by another researcher to ensure accuracy and consistency. In case of differences, negotiations were done to reach an agreement. In Table 3, the components of the TEA questionnaire and their encoded themes are illustrated with some related items.

**Table 2:** Semi-structured interview questions, their functions, and sources

Question	Function	Source
1. How can teachers’ past personal experiences affect their professional agency?	Iterational	Priestley et al. (2015). Teacher Agency: What Is It and Why Does It Matter?. In *Flip the System* (*pp. 134-148*). Routledge.
2. How can teachers’ past professional experiences affect their professional agency?	Iterational	
3. How can cultural resources such as ideas, values, beliefs, discourses, and dialogues facilitate or hinder teachers’ professional agency?	Practical-evaluative	
4. How can structural resources such as roles, relationships, power, and trust facilitate or hinder teachers’ professional agency?	Practical-evaluative	
5. How can material resources and physical environments facilitate or hinder teachers’ professional agency?	Practical-evaluative	
6. How can teachers’ short-term goals affect their professional agency?	Projective	
7. How can teachers’ long-term goals affect their professional agency?	Projective

**Table 3:** Initial components and retrieved themes in the TEA questionnaire

Component	Theme	Example
Iterational	Personal experiences Professional experiences Professional knowledge and skills Professional and personal beliefs and values rooted in past experiences	7. I exert my past personal and professional experiences to find practically pertinent courses of action in my class.
Practical-evaluative	Ideas, and discourses Social structures and relationships material resources Physical environment	13. Availability of teaching materials and resources can improve the quality of teachers’ teaching practice.
Projective	Short-term goals and long-term goals	29. I set goals to promote my students’ learning outcomes.

Putting the outcomes attained from the comprehensive review of the literature on teachers’ ecological agency together with the results of the semi-structured interviews conducted with 20 EFL teachers, a 40-item questionnaire was constructed which has been designed on a Likert scale ranging from 1 to 5. That is, strongly disagree=1, disagree=2, neutral=3, agree=4, and strongly agree=5. It is worth mentioning that some items were derived from the literature, whereas some other items were generated based on interviewees’ responses to the semi-structured interview questions.

Afterward, to evaluate the content validity of the questionnaire, a panel of 5 experts including 3 TEFL associate and assistant professors and 2 EFL teachers reviewed the items and confirmed the content validity of the initial draft of the questionnaire. Then the newly developed questionnaire consisting of 40 items (11 items for iterational, 17 items for practical-evaluative, and 12 items for projective component) was administered to 222 EFL teachers from various universities and institutes in Iran applying non-probability convenience sampling. All 222 participants answered all questionnaire items. Due to the Covid-19 pandemic, the researchers of the current study conducted an online questionnaire implementing the Google Forms platform and distributed it via email or other social media.

To calculate the reliability and construct validity of the newly developed questionnaire, it was subjected to Cronbach alpha, exploratory and confirmatory factor analysis through running SPSS. Additionally, structural equation modeling (SEM) was conducted to determine the path orientation of the underlying components of teacher ecological agency and their factor loadings.

### Design

**Fig. 2:**
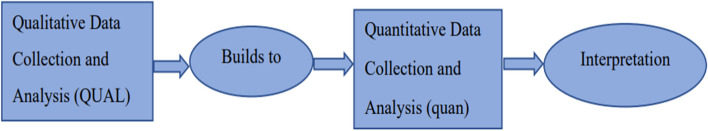
Exploratory sequential mixed methods (Cresswell, 2014, p.220)

The current study was conducted by applying an exploratory sequential mixed methods design. Generally, the major purpose of applying a mixed methods inquiry is to join qualitative and quantitative methods in various fashions and implement their strengths in a single inquiry. Exploratory sequential mixed methods research is a two-phase inquiry in which the qualitative data are gathered and analyzed in the initial phase. Based on the results of the first phase, then in the second phase, the quantitative data are gathered and analyzed. That is to say, for designing and developing a questionnaire, a three-phase process including examining the qualitative data, developing the evaluation inventory, and finally running the instrument to an adequate sample size should be followed (Cresswell, 2014). Figure 2 demonstrates this design.

## Results

This study was undertaken in order to explore the reliability, and validity, both exploratory and confirmatory, of the Teacher Ecological Agency (TEA) questionnaire. The TEA questionnaire had 40 items (Appendix 1) and was administered to 222 EFL teachers. The questionnaire had three components as follows: iterational (11 items), practical-evaluative (17 items), and projective (12 items).

### Cronbach’s alpha reliability indices

Table 4 displays Cronbach’s alpha reliability indices for the TEA questionnaire and its three components for the overall questionnaire, and the one after omitting items that had low or negative item-total correlations (Appendix 2). The results indicated that the reliability indices for the original questionnaire and the reduced one were higher than .70 which was considered as appropriate, as noted by Dörnyei and Taguchi (2009), who believed that Cronbach’s alpha value of .70 is the adequate reliability index for an instrument.

**Table 4:** Cronbach’s alpha reliability statistics of Teacher Ecological Agency questionnaire

	Alpha	*N* of items	Alpha	*N* of items
Iterational	.812	11	.875	9
Practical-evaluative	.884	17	.926	14
Projective	.863	12	.918	10
TEAQ	.858	40	.893	33

### Exploratory factor analysis

**Fig. 3:**
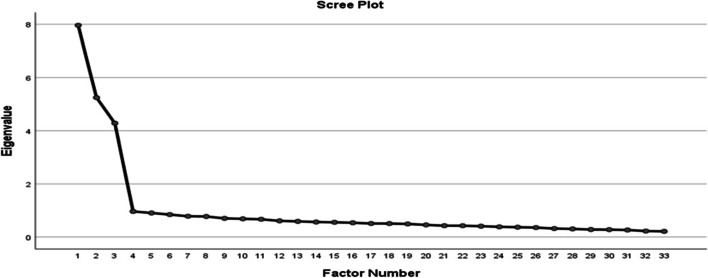
Scree plot for Teacher Ecological Agency questionnaire

An exploratory factor analysis (EFA) using principal axis factoring with varimax rotation (Appendix 3) was run using the reduced questionnaire to explore the underlying constructs of the 40 items of the TEA questionnaire. Based on the results of the scree plot (Fig. 3), it was decided to extract three factors as the underlying construct of the TEA questionnaire.

Table 5 displays the results of the KMO test of sample adequacy and Bartlett’s Test of Sphericity for the 33 items of the TEA questionnaire after omitting the nine items which did not load under their respective factors. The KMO index was .911, it was concluded that the present sample size was “marvelous” (Field, 2018, p. 1014) for running the EFA. The significant results of Bartlett’s Test (*χ*^2^ (528) = 3550.98, *p* < .05) indicated that the correlation matrix was factorable.

**Table 5:** KMO and Bartlett’s Test (after omitting items that did not load under their respective factors)

Kaiser-Meyer-Olkin Measure of Sampling Adequacy		.911
Bartlett’s Test of Sphericity	Approx. chi-square	3550.981
	df	528
	Sig.	.000

The EFA extracted three factors as the underlying constructs of the 33 items of the TEA questionnaire. This three-factor solution accounted for 48.38% of the total variances (Table 13, Appendix 4). And finally, Table 6 displays the factor loadings of the 33 items of the TEA questionnaire under the three extracted factors. The results indicated that all 14 items related to practical-evaluative loaded under the first factor. The 10 items measuring projective construct loaded under the second factor, and the 9 items related to iterational construct loaded under the third factor.

**Table 6:** Rotated Factor Matrix (after omitting items that did not load under their respective factors)

Item	Practical-evaluative	Item	Projective	Item	Iterational
Pra6	.750	Pro10	.762	Inter11	.756
Pra12	.735	Pro4	.757	Inter6	.749
Pra2	.728	Pro5	.744	Inter4	.701
Pra5	.720	Pro2	.737	Inter5	.697
Pra1	.719	Pro9	.729	Inter8	.646
Pra9	.700	Pro7	.723	Inter10	.631
Pra3	.690	Pro6	.719	Inter2	.605
Pra14	.689	Pro3	.706	Inter7	.580
Pra8	.682	Pro1	.696	Inter1	.572
Pra17	.667	Pro12	.643		
Pra4	.649				
Pra15	.622				
Pra10	.618				
Pra16	.582				

### Confirmatory factor analysis

A confirmatory factor analysis (CFI) was run to explore the underlying constructs of the TEA questionnaire. Before discussing the results, it should be noted that the CFI, besides the reliability of the instrument (Table 4), assumes univariate and multivariate normality. As displayed in Table 7, the values of skewness and kurtosis were within the ranges of +/− 2 (Bachman, 2005; Bae & Bachman, 2010). Thus, it can be concluded that the assumption of univariate normality was retained. The assumption of multivariate normality was probed through Mardia’s index. As noted by Khine (2013), the assumption of multivariate normality can rarely be retained in practice. He further proposed that Mardia’s index should be compared against the criterion of p*(*p*+1), where *p* stands for the number of items in a model; i.e., 33 in this study. Since Mardia’s index of −9.144 was lower than the criterion value of 1122; that is to say, 33*(33+1), it was concluded that the assumption of multivariate normality was also retained.

**Table 7:** Testing univariate and multivariate assumptions

Inter1	−0.04	−0.49	Pro6	0.02	−0.66	Pra8	0.19	−0.37
Inter7	−0.01	−0.49	Pro7	0.17	−0.62	Pra14	−0.17	−0.53
Inter2	0.16	−0.41	Pro9	0.05	−0.67	Pra3	0.01	−0.68
Inter10	−0.16	−0.50	Pro2	−0.02	−0.56	Pra9	−0.05	−0.75
Inter8	0.06	−0.68	Pro5	−0.06	−0.66	Pra1	−0.16	−0.41
Inter5	0.09	−0.72	Pro4	−0.18	−0.70	Pra5	−0.10	−0.40
Inter4	0.00	−0.56	Pro10	−0.02	−0.38	Pra2	−0.02	−0.77
Inter6	−0.17	−0.47	Pra16	−0.08	−0.44	Pra12	−0.15	−0.69
Inter11	0.06	−0.64	Pra10	0.02	−0.70	Pra6	0.04	−0.64
Pro12	−0.06	−0.29	Pra15	−0.25	−0.51	Mardia		−9.14
Pro1	0.07	−0.61	Pra4	−0.02	−0.54			
Pro3	−0.12	−0.64	Pra17	−0.02	−0.38			

**Fig. 4:**
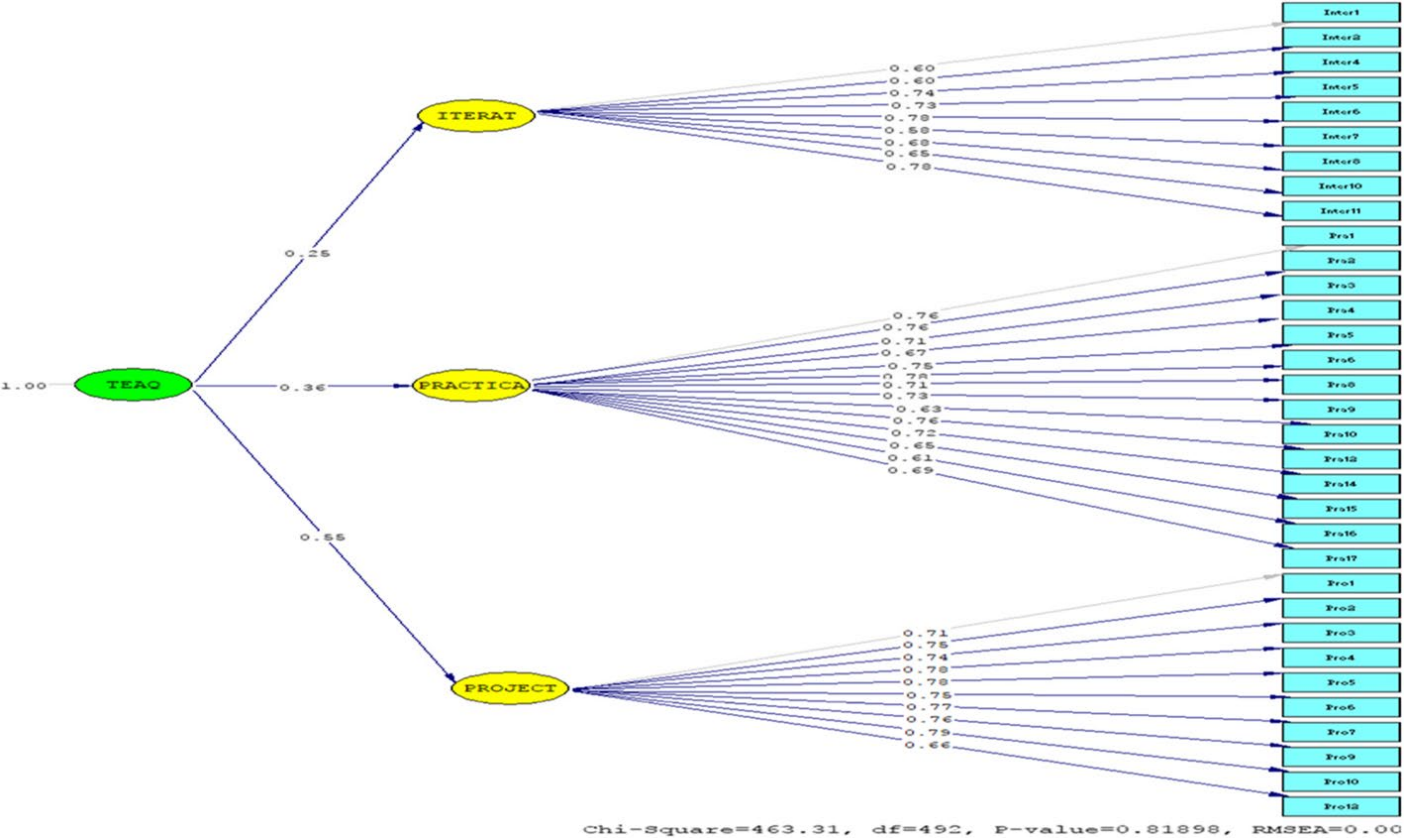
Structural equation model of Teacher Ecological Agency questionnaire

The model of the TEA questionnaire (Fig. 4) enjoyed good psychometric properties. The chi-square index was not significant (*χ*^2^ (492) = 463.31, *p* > .05). Its ratio over the degree of freedom; i.e., .941 was lower than 3. The RMSEA of .000, and its 90 % confidence intervals [.000, .015] were lower than .10. The PCLOSE fit of 1 was higher than .05. The SRMR of .043 was lower than .05. The GFI=.99, IFI=1.00, RFI=.95, NFI=.96, and CFI=1.00 were all higher than .90; and finally, the critical *N* value of 271.89 was higher than 200 indicating the adequacy of the present sample size (Table 8).

**Table 8:** Fit indices for final model

	TEA questionnaire	Criterion
Chi-Square	463.31	---
Degree of freedom	492	---
Sig.	.82	≥.05
Ratio	.941	<3
RMSEA	.000	≤.10
90 % CI for RMSEA	[.000, .015]	≤.10
PCLOSE	1.00	>.05
GFI	.99	≥.90
NFI	.96	≥.90
CFI	1.00	≥.90
RFI	.95	≥.90
SRMR	.043	≤.05
IFI	1.00	≥.90
CN	271.89	>200

## Discussion

In the present study, the researchers aimed to develop and validate a questionnaire to assess EFL teachers’ ecological agency. The major finding was that the Teacher Ecological Agency questionnaire enjoyed an acceptable index of reliability. Cronbach alpha reliability indices for iterational, practical-evaluative, projective, and overall ecological agency were respectively .812, .884, .863, and .858. Exploratory and confirmatory factor analyses confirmed three components of the TEA questionnaire including iterational, practical-evaluative, and projective which are all in compliance with the three dimensions of Priestley et al. (2015) ecological agency model as the underpinning theoretical foundation of this study. Each component would be discussed in details here.

The first component of the TEA questionnaire, iterational, consists of 11 items. This component mainly addresses teachers’ past personal and professional experiences. Numerous researchers support the profound effect of teachers’ experiences on their effectiveness of teaching practice and agency (Altan & Lane, 2018; Berger et al., 2018; Bukor, 2013; Irvine, 2019; Muhonen et al., 2021; Naraian & Schlessinger’s, 2018; Podolsky et al., 2019; Wang et al., 2021). For instance, Wang et al. (2021) found out that teacher agency construction was related to their past teaching experiences and the development of international pedagogies. Similarly, Altan and Lane (2018) indicated that significant experiences and dispositions in teachers’ lives affect their teaching practice. Likewise, Naraian and Schlessinger (2018) reported just one inclusive education teacher burdened himself with the unreasonable assignment of students of color in particular education. His agentic sense of direction toward a confluent perception of ability is derived from his own earlier learning experience as a colored male student. In addition, their study revealed that teacher agency for inclusive education may be promoted when teachers are exposed to different professional learning experiences.

The second component of the TEA questionnaire, practical-evaluative, with 17 items is broad and multifaceted dealing with various aspects such as cultural, structural, and material dimensions. The cultural dimension refers to teachers’ way of thinking, speaking, beliefs, and discourses both inner and outer speech. Considering the role of teachers’ beliefs in their agency, Biesta et al. (2015) stated that although it is claimed that teachers’ beliefs play a major part in their profession, a noticeable discrepancy between teachers’ personal values and beliefs and broader organizational discourses and cultures, as well as a relative absence of a coherent and rigorous professional perception of the educational purposes recommend that the enhancement of teacher agency does not simply depend on the beliefs that teachers introduce in their teaching practice but also calls for collective appreciation and development. Several studies (Baxter et al., 2021; Huang et al., 2021; Jones & Charteris, 2017; Körkkö, 2021; Leijen et al., 2020; Lyons et al., 2016; Naraian & Schlessinger, 2018; Ouyang et al., 2021; Yeh et al., 2021) support the effect of critical and collaborative reflection on teacher agency. Baxter et al. (2021), for example, conducted reflective circles to explore teachers’ reactions to definite teaching experiences to create novel practices that may lead to classroom change. Results of this study indicated improved shared support from peers and increased understanding of the values that affected teachers’ actions and reactions. By the same token, Huang et al. (2021) stated that collaborative teaching and taking part in school decision-making are associated with teaching for creativity. Naraian and Schlessinger (2018) also concluded that teachers’ agency for inclusive pedagogy did not merely originate from decontextualized teaching competencies developed in teacher education programs yet was considerably facilitated by collegial relationships and interactions with their students. Moreover, Lyons et al.’s (2016) study revealed that joint endeavors changed teaching from a solitary career to collaborative work, which significantly improved their resilience and motivation. Therefore, teachers’ professional agency is influenced by professional interactions. Professional agent teachers purposely consider their co-workers and students as a source for learning.

The structural dimension reflects social structures and relationships that contribute to teacher agency. Empirical studies (Chaaban et al., 2021; Karimpour et al., 2022; Vaitzman Ben-David & Berkovich, 2021; Wang & Zhang, 2021) revealed how social structures of institutions influence teachers’ professional agency. Karimpour et al. (2022), for instance, found that the top-down policies of institutions not only have a negative effect on teachers’ emotions, autonomy, self-efficacy, and identity but also impede teachers’ agentic enterprise. Wang and Zhang (2021) also highlighted that principal support had a significant impact on teachers’ professional skills and agency. Similarly, Chaaban et al. (2021) indicated that teachers’ capacity to enact professional agency was either reinforced or hindered because of various factors which varied between the public and private sectors in various educational contexts. Accordingly, teachers are more engaged dialogically with a new policy and exert agency in schools or institutions that develop and encourage horizontal relationships rather than a hierarchical structure (Priestley et al., 2015).

The material dimension deals with resources and the physical environment that promote or demote teacher agency. Several researchers (Abonyi et al., 2020; Huang et al., 2021; Schwab et al., 2020; Toom et al., 2017) explored the effect of materials, resources, and physical environment on teachers’ agentic practices. Schwab et al.’s (2020) study revealed that a higher perception of available resources results in the perception of inclusive teaching practices and satisfactory resources as a prerequisite for successful schooling. Similarly, Huang et al. (2021) suggested that available equipment and resources are connected with creative teaching. In addition, Abonyi et al. (2020) found that the availability of required teaching and learning resources reinforced the transfer of teachers’ learning. Conversely, the lack of adequate teaching and learning materials inhibited the efficient transfer of teachers’ professional development.

Subsequently, the practical-evaluative feature illustrates the need for teacher education policies to go beyond emphasizing teacher capability, which primarily concentrates on a teacher’s competence, and to take into account contextual circumstances that could either impede or enhance teachers’ performance. Additionally, assessments of a teacher’s capacity in the setting in which they deliver inclusive education should be implemented in teacher education programs. Hence, developing teachers’ agency is not limited to providing teachers with professional development programs. To address the social, cultural, and structural aspects of the educational system, a more comprehensive and systematic transformation is also required (Li & Ruppar, 2021).

Finally, projective with 12 items as the last component targets teachers’ short-term and long-term goals. Hemi et al. (2021) suggested that academic goal attainment is closely related to teachers’ goal perception. Moreover, they found that perceived peer goals act as a mediator between perceived teacher goals and learners’ achievement. Camp (2017) also conducted a study to find out how goal setting can foster teacher professional growth. The result of this study revealed some probable factors such as goal commitment and self-efficacy can contribute to or hamper goal attainment. Moreover, findings illustrated that teachers considered goal setting as a positive investment of time and an influential factor in improving their teaching quality. In effect, the projective dimension of the ecological agency model is conceptualized as a process of constant creative reconstruction of the future that includes applying earlier experiences to define goals and motives in order to pinpoint potential future barriers and determine practically and morally pertinent courses of action (Priestley et al., 2015).

Due to the nonexistence of ample studies in the field of teacher agency questionnaires which was the main gap in the literature, the researchers of the current study attempted to develop and validate an instrument for this purpose. The only questionnaire developed for assessing teacher agency by Leijen et al. (2021) is to evaluate student-teacher ecological agency in three domains: planning of teaching and learning activities, teaching diverse abilities students in the same class, and using ICT (Information and Communication Technology) in teaching. Although Leijen et al.’s questionnaire and the newly developed TEA questionnaire were both designed based on the ecological agency model proposed by Priestley et al. (2015), some differences were found between these two inventories. Leijen et al.’s questionnaire consists of three sections, planning teaching and learning activities, teaching diverse abilities students in the same class, and using ICT in teaching. Each section encompasses 10 items designed based on iterational, practical-evaluative, and projective dimensions. Therefore, the items of this instrument are limited to assessing teachers’ ecological agency in specific domains. However, the novelty of the TEA questionnaire relies on designing items in a way that is to be employed for various domains and purposes in different educational contexts.

## Conclusion

Doubtlessly teaching is a dynamic and multifaceted profession lending itself truly to evolution and modification in the pertinent areas. As a pivotal feature of this dynamic field, teachers are demanded to adapt to these changes and develop themselves continually. Teacher agency as a core factor to respond to the changes received a surge of attention in educational contexts (Tinn & Ümarik, 2021). Accordingly, designing and validating a potential evaluation inventory for assessing EFL teachers’ ecological agency lies at the heart of this attempt that resulted in the development of a questionnaire with a final version including 33 items loaded on three constructs namely iterative, practical-evaluative, and projective that were scored on a 5-point Likert scale ranging from 1 to 5 showing the extent the statement was estimated to be correct for the participant teachers. Applying three analytical techniques, exploratory factor analysis, confirmatory factor analysis, and structural equation modeling, the newly developed questionnaire indicated sound psychometric properties and can be utilized as an appropriate instrument to evaluate EFL teachers’ ecological agency.

Several stakeholders can take advantage of this newly developed instrument such as teachers, researchers, teaching practitioners, teacher educators, and policymakers. Rather than being a static and innate quality that someone possesses, agency is created through the interplay between iterational, practical-evaluative, and projective dimensions. Teacher agency is an important aspect of teacher professionalism, particularly in the neoliberal era where teacher education is criticized for undermining teachers’ potential to apply judgment and monitor their own teaching practice (Jones & Charteris, 2017). In this regard, recognizing the importance of agency, teachers will enact more changes and make principled decisions to promote the quality of their teaching. Moreover, by applying the TEA questionnaire, researchers and teaching practitioners can assess teachers’ level of agency, and teacher educators can provide them with learning programs and assignments related to various aspects of ecological agency and assist them to identify their agency formation. In addition, since teacher agency is a multifaceted concept, not only teachers’ beliefs, values, and personal and professional experiences but also the contextual and structural factors affect teachers’ agency. Therefore, policymakers have a key role in providing appropriate policies and infrastructures to empower teachers to act more agently in their classrooms.

As with the majority of studies, there are certain limitations in this study that should be taken into account. First, the small sample size in the current study could be regarded as the main limitation. Therefore, as far as generalizability is concerned, it is required to apply the newly developed TEA questionnaire with a larger sample size. In addition, due to the Covid-19 pandemic, not only having access to a large sample size was not feasible but also participants’ individual differences, their age, gender, experience, social, emotional, and cognitive background were not truly controlled. Moreover, the qualitative phase of this study was carried out by reviewing the related literature and conducting semi-structured interviews. Other researchers can replicate this study by applying various data collection instruments such as focused group discussions, documenting, and classroom observation to achieve a deeper understanding of the concept of ecological teacher agency. Finally, the TEA questionnaire was designed and developed based on Priestley et al.’s (2015) ecological model, and future studies can be conducted considering other approaches to teacher agency.

## Figures and Tables

**Fig. 5:**
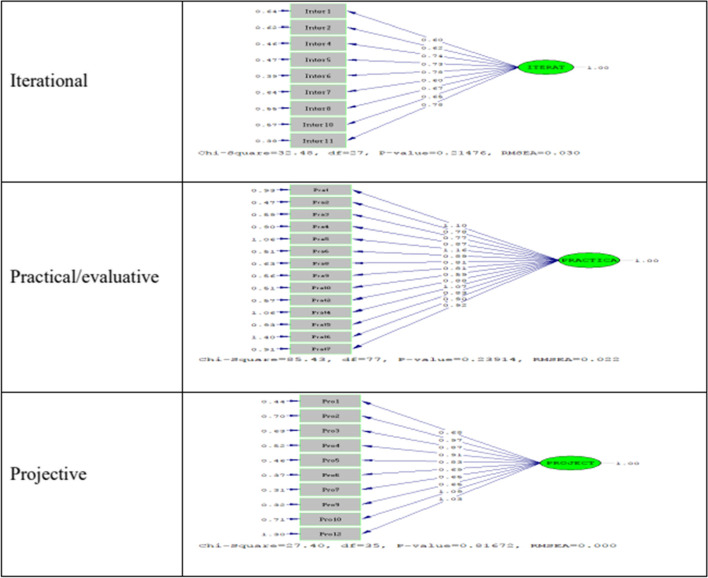
Measurement Models of Components of Teacher Ecological Agency Questionnaire

## Data Availability

Data is available for submission if required through anonymous email.

## Appendix 1

Table 9

**Table 9:** Teacher Ecological Agency (TEA) questionnaire

	Statements	1	2	3	4	5
**Iterational**						
1	I draw upon my experiences in past as a learner to take principled decisions about my teaching practice in class.	1	2	3	4	5
2	I take advantage of my earlier experience of teaching to deal with everyday teaching challenges.	1	2	3	4	5
3	Personal beliefs and values that I bring to my class affect my teaching-related activities.	1	2	3	4	5
4	My professional knowledge and skills play a crucial role in the type of teaching actions I take in my class.	1	2	3	4	5
5	I attempt to identify and assign past patterns of behavior to deal with current dilemmas in my teaching profession.	1	2	3	4	5
6	I rely on my past personal and professional experiences to predict probable upcoming constraints.	1	2	3	4	5
7	I exert my past personal and professional experiences to find practically pertinent courses of action in my class.	1	2	3	4	5
8	I draw upon my day-to-day teaching experiences to make necessary changes in my teaching practice.	1	2	3	4	5
9	teachers who have experienced other professions solve their daily teaching problems more effectively.	1	2	3	4	5
10	Teachers’ schooling and education have an important role in the development of their potential to innovate.	1	2	3	4	5
11	I employ my personal beliefs and experiences to respond strategically to the challenging situations imposed by the school policy.	1	2	3	4	5
**Practical-evaluative**						
12	Teachers’ professional environment affects their teaching-related activities.	1	2	3	4	5
13	The availability of teaching materials and resources can improve the quality of teachers’ teaching practice.	1	2	3	4	5
14	I reflect critically to find the best solutions for the problems I face in my class.	1	2	3	4	5
15	The collaborative reflection process through which teachers engage dialogically with their colleagues impacts their teaching practice in a positive way.	1	2	3	4	5
16	School principals have an important role in establishing trust and providing facilitation for teachers.	1	2	3	4	5
17	School managers influence teachers’ interpretations of the anticipated learning outcomes.	1	2	3	4	5
18	School principals affect teachers’ understanding of which of their strategies contribute to the achievement of the desired learning outcome.	1	2	3	4	5
19	School leaders should help teachers set their goals to develop their students’ learning.	1	2	3	4	5
20	School authorities should provide teachers with professional development programs to promote their knowledge and skills.	1	2	3	4	5
21	Teachers can make appropriate decisions and changes in their classes when they are given the voice and power to act.	1	2	3	4	5
22	The existence of robust professional discourse about education enhances the actions teachers take in their classes.	1	2	3	4	5
23	Access to wider professional discourses about teaching should be an integral part of teacher education.	1	2	3	4	5
24	Habitual ways of thinking about education hinder teachers to make informed decisions and take principled actions.	1	2	3	4	5
25	Social relationships between the teacher, school manager, and colleagues impact teachers’ decisions and actions.	1	2	3	4	5
26	Teachers cope more successfully with challenges in schools and educational contexts that develop effective social structures and encourage social relationships.	1	2	3	4	5
27	The educational context that encourages innovation and a questioning mindset contributes teachers to make more informed decisions and take action in their classes.	1	2	3	4	5
28	Teachers can make righteous decisions and changes in an educational setting that encourages horizontal social relationships rather than hierarchical social relationships.	1	2	3	4	5
**Projective**						
29	I set goals to promote my students’ learning outcomes.	1	2	3	4	5
30	I do my best to maximize my students’ potential.	1	2	3	4	5
31	I think teaching goals should not be limited to getting through the syllabus.	1	2	3	4	5
32	The utmost goal of education is to enable students to function effectively in society.	1	2	3	4	5
33	I set clear short-term goals in teaching to achieve the best results.	1	2	3	4	5
34	I set clear long-term goals to attain the desired outcome.	1	2	3	4	5
35	I try to keep my students interested and engaged.	1	2	3	4	5
36	Students’ welfare and development are important to me.	1	2	3	4	5
37	I attempt to peruse my goals strategically in my class.	1	2	3	4	5
38	I am motivated enough to do my best for my students.	1	2	3	4	5
39	I am interested in introducing new forms of pedagogy to promote the quality of education.	1	2	3	4	5
40	I try to maintain a normal desirable state in my class.	1	2	3	4	5

1=never; 2=only occasionally; 3=sometimes; 4=usually; and 5=always or almost always

## Appendix 2

Table 10 displays the item-total correlations for the 40 items of the TEA questionnaire. Before discussing the results, it should be noted that an appropriate item should have an item-total correlation of .30 or higher, as noted by Pallant (2016), and Field (2018). The results indicated that the following items had either negative item-total correlation or had indices lower than .30; projective (Items 3 and 9), practical-evaluative (Items 7, 11, and 13), and projective (Items 8, and 11).

**Table 10:** Item-total correlations

	Corrected item-total correlation			
	Practical-evaluative	Projective	Iterational	
Item1	.686	.632	.511	
Item2	.692	.691	.533	
Item3	.634	.667	**.039**	
Item4	.613	.700	.628	
Item5	.672	.711	.651	
Item6	.727	.666	.675	
Item7	**.055**	.694	.520	
Item8	.639	**.046**	.577	
Item9	.682	.665	**.018**	
Item10	.593	.715	.570	
Item11	**-.013**	**-.012**	.685	
Item12	.687	.603		
Item13	**.063**			
Item14	.641			
Item15	.598			
Item16	.546			
Item17	.630			

Table 11 displays the item-total correlations for the 33 items of the TEA questionnaire after removing the items which had negative or low item-total correlations. The results showed that all items had item-total correlations higher than .30.

**Table 11:** Item-total correlations (after omitting items with low or negative item-total correlations)

	Corrected item-total correlation			
	Practical-evaluative	Projective	Iterational	
Item1	.696	.665	.538	
Item2	.701	.704	.557	
Item3	.662	.684	.655	
Item4	.624	.721	.648	
Item5	.695	.718	.692	
Item6	.725	.690	.537	
Item7	.659	.702	.609	
Item8	.676	.700	.592	
Item9	.589	.731	.703	
Item10	.707	.615		
Item11	.666			
Item12	.598			
Item13	.567			
Item14	.645			

## Appendix 3

Before discussing the results, it should be noted that EFA can be run through two types of rotation methods; orthogonal and oblique. Orthogonal rotation assumes that factors underlying observed variables are not correlated, whereas oblique rotation assumes that factors are correlated. Field (2018) believes that one can hardly find any two constructs that are not correlated in humanities. Consequently, he proposed the oblique rotation in such researchers. However, there is a statistical technique to decide which rotation method should be used. The researcher consulted the “Factor Correlation Matrix” (Table 12). This matrix shows the correlations among the extracted factors. Ignoring the ones on the diagonal, if the correlation coefficients are higher than +/− .32 (Dagdag et al., 2020; Grande, 2016), oblique rotation should be used. Since all correlation coefficients were lower than +/− .32, varimax rotation was employed to explore the underlying constructs of the TEA questionnaire.

**Table 12:** Component correlation matrix

Component	1	2	3	4	5	6	7	8
1	1.000							
2	.154	1.000						
3	.060	.100	1.000					
4	−.004	−.003	.044	1.000				
5	.022	−.019	−.012	−.064	1.000			
6	−.029	−.004	−.047	.021	.002	1.000		
7	−.084	−.008	−.044	.017	−.015	.028	1.000	
8	.041	−.036	−.079	−.015	.026	−.072	.014	1.000

## Appendix 4

Table 13

**Table 13:** Total variance explained (after omitting items that did not load under their respective factors)

Factor	Initial eigenvalues			Extraction sums of squared loadings			Rotation sums of squared loadings		
	Total	% of variance	Cumulative %	Total	% of variance	Cumulative %	Total	% of variance	Cumulative %
1	7.962	24.127	24.127	7.459	22.604	22.604	6.630	20.091	20.091
2	5.244	15.891	40.018	4.754	14.405	37.008	5.322	16.127	36.218
3	4.283	12.978	52.995	3.753	11.373	48.382	4.014	12.164	48.382
4	.966	2.928	55.923						
5	.908	2.750	58.674						
6	.847	2.568	61.242						
7	.786	2.382	63.623						
8	.777	2.354	65.977						
9	.706	2.138	68.116						
10	.689	2.087	70.202						
11	.671	2.034	72.236						
12	.612	1.854	74.090						
13	.593	1.797	75.887						
14	.569	1.724	77.611						
15	.558	1.691	79.302						
16	.540	1.636	80.938						
17	.513	1.554	82.492						
18	.511	1.548	84.041						
19	.496	1.504	85.545						
20	.460	1.393	86.938						
21	.431	1.305	88.243						
22	.429	1.300	89.543						
23	.412	1.247	90.790						
24	.388	1.176	91.966						
25	.374	1.134	93.100						
26	.360	1.092	94.193						
27	.323	.980	95.173						
28	.310	.938	96.111						
29	.286	.866	96.978						
30	.282	.854	97.831						
31	.269	.815	98.647						
32	.229	.694	99.341						
33	.217	.659	100.000						

## Appendix 5

Table 14 displays the fit indices for the three measurement models displayed in Fig. 5 (below). The results indicated that all three measurement models enjoyed good fit indices as discussed below;

A: The chi-square results were non-significant for the iterational (χ^2^ (27) = 32.48, *p* > .05), projective (χ^2^ (35) = 27.42, *p* > .05) practical-evaluative model (χ^2^ (77) = 85.43, *p* > .05) models. The ratios of chi-square over their degree of freedom were lower than 3 for iterational (1.20), practical-evaluative (1.10), and projective (.697).

B: The root mean square of error approximation (RMSEA) indices were lower than .10 which is considered as acceptable (Byrne, 2010). The RMSEA for three models were .030, .022 and .000. The 90 percent confidence intervals for RMSEA were also lower than .10; iterational [.000, .063], practical-evaluative [.000, .046], and projective [.000, .034].

C: The probability of close fit for all three models was higher than .05 which showed a good fit; i.e. .81, .98, and 1.00.

D: The standardized root mean residuals for three models were lower than .05; i.e. .033, .035, and .025, respectively for iterational, practical-evaluative, and projective models.

**Table 14:** Fit indices for measurement models

	Iterational	Practical-evaluative	Projective	Criterion
Chi-square	32.48	85.43	27.42	---
Degree of freedom	27	77	35	---
Sig.	.21	.24	.82	≥.05
Ratio	1.20	1.10	.697	<3
RMSEA	.030	.022	.000	≤.10
90 % CI for RMSEA	[.000, .043]	[.029, .065]	[.000, .062]	≤.10
PCLOSE	.81	.98	1.00	>.05
GFI	1.00	1.00	1.00	≥.90
NFI	.98	.98	.99	≥.90
CFI	1.00	1.00	1.00	≥.90
RFI	.98	.98	.99	=>.90
SRMR	.033	.035	.025	<=.05
IFI	1.00	1.00	1.00	=>.90
CN	320.54	279.85	463.54	>200

E: The goodness of fit (GFI), normed fit (NFI), comparative fit (CFI), incremental fit (IFI), and relative fit (RFI) indices were all higher than .90. All these results supported the fit of the models.

F: And finally; the present sample size was adequate for running CFI. The critical N (CN) indices were all higher than 200.
